# MEETING SOCIAL NEEDS TO IMPROVE HEALTH OUTCOMES: PARTNERSHIPS BETWEEN COMMUNITY-BASED ORGS AND HEALTH CARE

**DOI:** 10.1093/geroni/igz038.1849

**Published:** 2019-11-08

**Authors:** Traci L Wilson, Suzanne R Kunkel, Jane Straker, Marisa Scala-Foley, Elizabeth Blair

**Affiliations:** 1 Miami University Scripps Gerontology Center, Oxford, Ohio, United States; 2 National Association for Area Agencies on Aging (n4a), Washington, D.C., United States

## Abstract

Unmet social needs negatively affect individual and population health, and better integration of community-based supports and health systems is a promising approach to improve health outcomes and avoid unnecessary health care use. Community-based organizations (CBOs) such as Area Agencies on Aging (AAAs) and Centers for Independent Living (CILs), as providers and coordinators of social services, are well-positioned within their communities to coordinate care and provide for unmet social needs. Partnerships between CBOs and health care entities have clear potential to improve health care outcomes while also reducing expenditures. This paper will present a cross-sectional analysis of a national survey of AAAs, CILS, and other CBOs at two time points (2017: n=593; 2018: n=763) to understand the extent, type, and evolution of CBO engagement with health care providers. In addition, longitudinal analysis (n=374) shows movement at the organization level: 33% of organizations who did not have a contract at T1 but were pursuing one had achieved a contract by T2. This presentation will: describe details of the services delivered, contracting arrangements, and populations served under CBO/health care contracts, as well as challenges experienced by CBOs; examine differences by state and organizational structure; and discuss the implications of state policy on integrated care and contracting.

